# Multicenter Randomized Trial of 10-French versus 11.5-French Plastic Stents for Malignant Biliary Obstruction

**DOI:** 10.1155/2013/891915

**Published:** 2013-05-02

**Authors:** Mihir S. Wagh, Mario de Bellis, Evan L. Fogel, James T. Frakes, John F. Johanson, Tahir Qaseem, Douglas A. Howell, Glen A. Lehman, Stuart Sherman

**Affiliations:** ^1^Division of Gastroenterology, University of Florida, 1600 SW Archer Road, HD 602, Gainesville, FL 32610, USA; ^2^Division of Gastroenterology/Hepatology, Indiana University Medical Center, Indianapolis, IN 46202, USA; ^3^Rockford GE Associates, Rockford, IL 61107, USA; ^4^Division of Gastroenterology, Maine Medical Center, Portland, ME 04102, USA

## Abstract

*Background*. There is little prospective data on whether bigger plastic stents are better for patients with malignant biliary obstruction with jaundice. *Goals*. Multicenter prospective study to compare technical success, clinical response, stent occlusion, and patient survival in patients with malignant biliary obstruction randomized to 10-French or 11.5-French plastic stent. *Study*. Patients with malignant biliary obstruction were randomized to 10-French or 11.5-French biliary stents. Patients were prospectively assessed for stent occlusion, stent-related interventions, hospital stay, and change in bilirubin. Main outcome measurements included technical success, clinical response, rates of stent occlusion, and survival. *Results*. 234 patients (47 hilar and 187 common bile duct strictures) were randomized. Outcomes were similar for the 10-French and 11.5-French groups (technical success 99.1% versus 97.4%, *P* = 0.37). Overall, median stent survival was 213 days, but there was no statistically significant difference in stent survival between 10-French and 11.5-French stents (149 versus 258 days, *P* = 0.16). Stent survival was significantly longer when placed for common bile duct versus hilar strictures (231 versus 115 days, *P* = 0.049). *Conclusions*. The theoretical advantage of improved bile flow for the 11.5-French stent does not translate into more prolonged patency, better clinical response, and longer patient survival than the 10-French stent.

## 1. Introduction

 The majority of patients presenting with symptoms of malignant biliary obstruction are considered unsuitable for surgery because of locally advanced or metastatic disease or poor performance status. Palliative biliary stenting at ERCP is frequently the only planned therapy. Endoscopic biliary stenting was first described by Soehendra and Reynders-Frederix [1] in 1980 and since then has become the preferred method to relieve jaundice and improve quality of life for patients with advanced malignant biliary obstruction [2–5]. Several studies have shown that this technique is associated with fewer complications and lower costs than surgical bypass or percutaneous drainage [6–10]. 

 The major limitation to long-term biliary stenting is stent occlusion which particularly affects plastic stents whose average patency is 4 months for the 10 French (Fr) polyethylene stents [11, 12]. The most commonly used plastic stent is the straight polyethylene stent with distal and proximal end holes and an adjacent side hole at its tip [13].

 The most cost effective strategy to prolong stent patency is the use of larger stent diameter. However, while in some studies stent patency was significantly longer for large-diameter plastic stent rather than small diameter stents, other investigators found no prolongation of stent patency and noted more complications when larger stents were used [14–19]. Another approach to prolong stent patency has been the use of expandable metal stents whose large diameter (30 Fr) ensures a long term patency rate which is about twice as long as the 10 Fr plastic biliary stent [20–27]. Though recent studies have shown the increasing role of expandable metal stents for initial biliary decompression of patients with malignant biliary obstruction, and despite the improved patency of metal stents, plastic stents are still widely used all over the world for treatment of malignant bile duct obstruction being especially cost effective in patients with estimated survival of less than 4 months and in countries, with low-cost ERCP. Thus while expandable metal stents may be the near standard in wealthy countries it is not that way in many other countries.

 Given all the above, the standard polyethylene plastic stent is still often the first choice for endoscopic biliary stenting of patients with established or suspected malignant biliary obstruction (though expandable metal stents may be initially placed in these patients). The question of whether “bigger is better” still has not been answered. It remains controversial whether the potential advantage of improved bile flow for the 11.5 Fr stent is clinically significant and outweighs the disadvantage of using these more cumbersome stents. We conducted a multicenter prospective randomized study that compared the 10 Fr and 11.5 Fr polyethylene (plastic) biliary stents in patients with suspected or proven malignant biliary obstruction.

## 2. Methods

Patients with suspected or proven malignant biliary obstruction who were unresectable or inoperable and were undergoing ERCP were candidates for the study. This was a multicenter randomized trial conducted at 3 institutions: Indiana University Medical Center, Maine Medical Center, and Rockford GE Group. Approval for the study from the Institutional Review Board of each institution was obtained. A written and informed consent for the procedure with details about the stenting procedure and the two different plastic biliary stents used was obtained in writing from all patients.

### 2.1. Exclusion Criteria

Patients were excluded from the study if (a) they had previously undergone biliary stenting, (b) surgery was planned, (c) a guidewire could not be passed through the stricture, (d) they had an expected survival of <3 months, or (e) there was impending duodenal obstruction. This study compared the ability to place a 10 Fr versus 11.5 Fr biliary stent, and hence patients in whom a guidewire could not be passed above the stricture were excluded as neither type of stent could be placed in these cases. Also, patients with expected survival of <3 months were excluded to allow assessment of stent occlusion which would not be possible if the patient expired within 3 months. 

### 2.2. Procedure/Technique

After cannulation of the main bile duct, a cholangiogram was obtained to define the location and the extent of the biliary stricture. A guidewire was then passed through the stricture and, after securing access to the proximal biliary tree with the wire, more complete intrahepatic filling was obtained when felt clinically necessary. A biliary sphincterotomy was performed at the discretion of the endoscopist performing the procedure. The biliary stricture was then sampled for tissue and/or dilated at the discretion of the endoscopist. The patient was randomized to a 10 Fr or an 11.5 Fr plastic biliary stent following guidewire advancement upstream to the stricture using random computer-generated numbers and sealed opaque envelopes. The biliary stent chosen was placed using standard techniques. Hilar and common bile duct (CBD) strictures were randomized separately. Patients with hilar strictures had only one stent placed on the more obstructed/dilated side and based on pre-ERCP radiographic findings of intrahepatic duct dilation and hepatic lobe atrophy. Efforts were made to avoid contrast filling and manipulation of liver segments that were not going to be stented. Stents were not changed prophylactically. When a patient developed signs and symptoms suggestive of stent occlusion, the stent was changed on an emergent basis. Stent occlusion was confirmed by the 10 cm water column test [28].

### 2.3. Follow-Up

Patients were followed till death or first stent-related intervention by telephone interview or clinic visit at 1- to 2-month intervals after stent placement to assess for symptoms of stent occlusion and to determine the clinical response rate. The 10 Fr and 11.5 Fr stent groups were compared for technical success rate of stent deployment, clinical response rates, rates of stent occlusion, number of stent-related interventions and hospital days, and patient survival. Stent obstruction from various causes (occlusion, migration, or tumor overgrowth) was separately assessed as well. 

### 2.4. Statistical Analysis

The statistical analysis was performed using the log rank test, the Wilcoxon test, and the chi-square test. Pearson's chi-square test was used to compare categorical variables between the groups, and Fisher's exact test was used when cell frequencies were low. For continuous variables, a two-sample *t*-test or Wilcoxon's test was used to compare the groups after checking the assumption of normality and homoscedasticity. Regression analysis using baseline bilirubin as a covariate was used to reassess the effect of stent on bilirubin change. The interaction between stent group and baseline bilirubin was tested but was not significant. Diagnostics for model lack of fit and presence of outliers suggested 3 outliers and influential points. The regression model was run with and without the outliers. Statistical analyses were performed using the statistical software package SAS version 9.1 (SAS Institute, Cary, NC). Estimates of patient survival and stent survival were analyzed by the Kaplan-Meier method. Patients who died before a reintervention was required (i.e., stent exchange) were censored from the analyses of stent survival. In all the analysis performed a *P* value less than 0.05 was considered statistically significant. 

 Our prior experience indicated a median stent survival of 5 months for the 10 Fr stent. The estimated number of patients to gain 83% power (*P*-sided alpha = 0.05) to detect a 50% increase in the median stent survival to 7.5 months was 100 patients in each group.

## 3. Results

**Figure 1:**
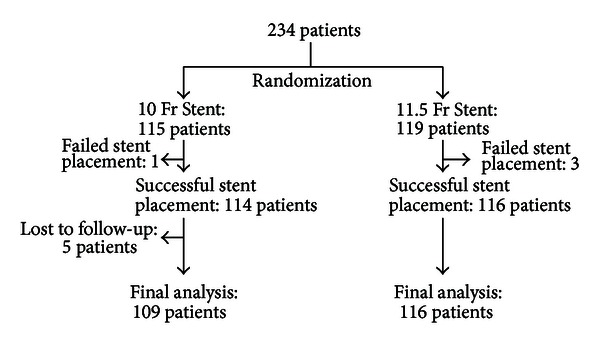


 During a 3-year period 234 patients with suspected or proven malignant obstructive jaundice underwent ERCP for biliary decompression and were randomized to a 10 Fr or an 11.5 Fr plastic biliary stent (Figure 1). 115 patients received a 10 Fr stent and 119 were stented with an 11.5 Fr stent. There were 133 males and 101 females (median age 68 years). 80% (187) of patients had a common bile duct stricture and 20% (47) a hilar stricture. Tumor types included pancreatic cancer (56%), cholangiocarcinoma (22%), metastatic tumors (13%), gallbladder cancer (4%), and other (5%). The two stent groups were similar with regard to the age, gender, stricture location, and stent diameter placed at center (Table 1). 

 Stents were successfully placed in 114/115 patients (99.1%) in the 10 Fr group and 116/119 patients (97.4%) in the 11.5 Fr stent group (*P* = 0.37). Placement of 11.5 Fr biliary stents did not require larger biliary sphincterotomies or more aggressive dilation of the stricture. Five patients, who received a 10 Fr stent, were lost to follow-up. Therefore, the final analysis of the data was performed on a total of 225 patients (109 with 10 Fr stent and 116 with 11.5 Fr stent). The median follow-up was 143 days for the 10 Fr stent group and 159 days for the 11.5 Fr stent group.

 In the 225 patients stented, median overall stent survival was 213 days with 83 reinterventions (events) performed for stent exchange. However, stent survival was not significantly influenced by the stent size. The median patency for the 11.5 Fr stents was 258 days, while the 10 Fr stents stayed patent for 149 days (*P* = 0.16). When stent survival was related to the location of the stricture, it was found that the overall median stent patency was significantly longer for a CBD stricture (231 days) than for a hilar stricture (115 days, *P* = 0.049). There was a trend toward an increased stent survival for 11.5 Fr stents (311 days) compared to 10 Fr stents (149 days) placed for CBD strictures (*P* = 0.071), but this difference did not reach statistical significance. No statistically significant difference in stent survival was found between 11.5 Fr and 10 Fr stents when stent survival was examined for a hilar stricture location (*P* = 0.93). 

Table 2 shows the outcomes in both groups. The decrease in total bilirubin from baseline was 10.6 mg/dL in the 11.5 Fr group and 8.7 mg/dL in the 10 Fr group (*P* = 0.03). However, the baseline bilirubin prior to stent placement was 12.8 mg/dL and 10.8 mg/dL in the two groups, respectively (*P* = 0.03). To make valid comparisons between the 2 groups, the effect of stenting was reassessed and tested after adjusting for baseline bilirubin in a regression model. The model suggested a better fit for the data, with no stent effect. The bilirubin decrease (10.6 mg/dL in the 11.5 Fr group and 8.7 mg/dL in the 10 Fr group) was no longer significant (*P* = 0.077) after adjusting for the difference in baseline bilirubin. Stent-related hospital days to treat complications (i.e., cholangitis), stent-related interventions, frequency of death before stent occlusion, and patient survival were similar for the two groups. Stent obstruction occurred in 77 patients (38 (32%) in the 11.5 Fr group and 39 (34%) in the 10 Fr group) (Tables 2 and 3). More than one cause for stent obstruction (occlusion, migration, and tumor overgrowth) was seen in some patients.

Table 4 shows that there was no significant difference in stent obstruction when comparing 11.5 Fr and 10 Fr stents placed for CBD obstruction (27% versus 30%, *P* = 0.64) and when comparing these two stents placed for hilar lesions (50% versus 48%, *P* = 0.88).

 The incidence of early (<30 days) stent complications was similar for the two groups (Table 5) but this study was not powered to detect this. There were 13 early (<30 days) deaths (11%) in the 11.5 Fr stent group and 7 (6%) in the 10 Fr stent group (*P* = 0.19). Stent occlusion occurred in 4 (3%) patients with 11.5 Fr stents and in 6 (5%) patients with 10 Fr stents (*P* = 0.48). Stent migration rate was 2% in each of the two groups. There was no difference in rates of tumor overgrowth. 

## 4. Discussion

Patients with malignant biliary obstruction often have an unresectable tumor and/or are unfit for surgery. These patients require palliation of the obstructive jaundice and can be managed by surgery, endoscopy, or interventional radiology [6–10]. Since the initial report [1], endoscopic stenting has become the preferred method for palliation of patients with malignant obstructive jaundice and has been shown to improve the quality of life of these patients [4, 5, 29]. A recent meta-analysis concluded that both surgery and endoscopic stenting are effective for the palliation of malignant obstructive jaundice [30]. Patients undergoing endoscopic stenting have lower 30-day mortality and less early complications than surgical bypass but require repeat ERCP to exchange the plastic stent for occlusion [7–10, 30]. Ten-French plastic stents usually become obstructed after 4 months [11, 12].

 Stent occlusion is an important clinical problem that has been studied extensively in an attempt to improve stent patency. Since deposition of sludge in the stent is not preventable, the simplest and most effective approach to prolonging stent survival is to use large diameter stents. Rey and colleagues noted that by increasing the internal stent diameter by 0.2 mm the rate of the bile flow increased 300% [31]. Zimmon and Clemett reported good palliation of obstructive jaundice with single/multiple 5 Fr stents, but 38 episodes of cholangitis occurred in 22 patients (among the 162 patients stented) [32]. High rates of cholangitis (40%) were reported by Kiil and colleagues who used single 7 Fr stents for biliary decompression [33]. These stents had a relatively low patency rate with stent occlusion occurring at a median of 49 days after the initial placement. Insertion of multiple small-diameter stents as suggested by Zimmon and Clemett may improve the overall patency rate [32]. However, the flow capacity of two 5 Fr stents is nearly 10 times lower than that of a single 10 Fr stent [14]. These authors calculated that the flow capacity of a 10 Fr stent (103 mL/min) is 270% greater than that of an 8 Fr stent (41 mL/min) and recommended the use of at least a 10 Fr stent for endoscopic palliation of malignant biliary obstruction. Maillot and colleagues obtained similar results in a porcine model [34]. Siegel and colleagues reported that the patency rate of 12 Fr stents was longer than that of 10 Fr stents (190 versus 150 days) and recommended routine use of these large stents [15]. However, three retrospective studies failed to confirm that stents larger than 10 Fr have any significant advantage in the palliative treatment of patients with malignant biliary strictures compared with the 10 Fr stent [17, 18, 35, 36]. 

The question whether bigger is definitively better for a plastic stent has clinical importance since the plastic stent often is the first choice for the palliation of patients with malignant biliary obstruction, despite the introduction of metal stents into clinical practice [20, 21, 23, 26, 27]. Despite the improved patency of metal stents, plastic stents are still widely used for treatment of malignant bile duct obstruction, being especially cost effective in patients with estimated survival of less than 4 months and in countries with low-cost ERCP. Thus while expandable metal stents may be the near standard in wealthy countries it is not that way in many other countries.

 In this multicenter prospective randomized trial we evaluated the patency rates for 10 Fr and 11.5 Fr stents placed at ERCP to drain patients with malignant biliary obstruction. To our knowledge, this is the first prospective randomized trial addressed to answer the question whether bigger is better for plastic biliary stents. In our study, the technical success rate of stent placement, stent occlusion rate, stent-related interventions and hospital days, and the patients' survival were not statistically different for the 10 Fr and 11.5 Fr stent groups (Table 2). 

 Clinical response rate to stenting as assessed by reduction in bilirubin was different in the two groups. The decrease in total bilirubin from baseline was 10.6 mg/dL in the 11.5 Fr group and 8.7 mg/dL in the 10 Fr group (*P* = 0.03). However, there was a difference in the baseline bilirubin prior to stent placement in each group. After adjusting for this difference in baseline bilirubin, the decrease in bilirubin (10.6 mg/dL in the 11.5 Fr group and 8.7 mg/dL in the 10 Fr group) was no longer significant (*P* = 0.077). Also, from a practical standpoint, the numerical bilirubin value may not be very important in patient management as long as biliary stenting is able to alleviate jaundice. Hence the minimal difference in reduction in bilirubin after placement of 11.5 Fr or 10 Fr stents may not have much clinical significance.

 Our data on stent patency are similar to those reported by Kadakia and Starnes [17] although we found a slight trend toward stent survival for the 11.5 Fr stent. Matsuda et al. reported no significant difference in median stent survival for 10 Fr and 11.5/12 Fr stents [18]. Dowsett and colleagues reported no significant difference in stent survival between 10 Fr and 12 Fr stents [35]. However, the 12 Fr stents were more difficult to insert with a technical success rate of only 51% when compared to 98% for 10 Fr stents. In our study, the success rate of stent insertion was 99.1% for 10 Fr and 97.4% for 11.5 Fr stents (*P* = 0.37). Similar results were reported by Kadakia and Starnes who successfully placed a 10 Fr stent in 85% of cases and an 11.5 Fr stent in 79%. We noted that 11.5 Fr stents were somewhat more difficult to advance through the stricture although the time to stent placement was not measured. 

 In our study, a trend for improved patency rates was seen for the 11.5 Fr stent for common bile duct strictures. The 11.5 Fr stents had a median survival of 258 days while the 10 Fr stents stayed patent for a median of 149 days. However, this marked numerical difference was not statistically significant (*P* = 0.071), and there was no reduction in the number of stent-related interventions and hospital days for stent dysfunction. Although the sample size was small, there was no improvement or trend toward improvement in the stent patency rates for 11.5 Fr stents placed for hilar strictures (median patency of 112 days versus 115 days, respectively, for the 11.5 Fr and 10 Fr stents. *P* = 0.93). Stents placed for hilar obstruction in our series occluded faster than stents placed for more distal obstruction. Similarly, Dowsett et al. reported that stent changes were more frequent in high than in low (below cystic duct insertion) (30% versus 20%) common duct obstruction [35]. This is probably related to the longer stents required for proximal strictures and the slower flow dynamics [14].

The incidence of early (<30 days) stent complications was similar for the two groups, but this study was not powered to detect this. Similarly, there was no statistically significant difference in complication rates (*P* = 0.87) between 10 Fr and 11.5 Fr stent groups in the series reported by Kadakia and Starnes [17]. 

## 5. Conclusion

Our data suggests that the theoretical advantages of improved bile flow and long-term stent patency for the 11.5 Fr plastic stent are not seen clinically. However, the 11.5 Fr stent may be more effective than the 10 Fr stent in providing longer periods of drainage in common bile duct obstruction. This finding did not translate into a reduction in stent-related interventions or stent-related hospital days. There appears to be no advantage to decompressing a malignant biliary stricture with the more difficult-to-place 11.5 Fr stent. 

## Figures and Tables

**Table 1:** Demographic data: frequencies by stent size.

Variable	10 Fr (*n* = 115)	11.5 Fr (*n* = 119)	*P* value
Age			0.736
65	36.5% (42)	38.7% (46)	
>65	63.5% (73)	61.3% (73)	
Center			0.740
IU	60.9% (70)	62.2% (74)	
MMC	31.3% (36)	27.7% (33)	
RGC	7.8% (9)	10.1% (12)	
Gender			0.924
Female	43.5% (50)	42.9% (51)	
Male	56.5% (65)	57.1% (68)	
Stricture location			0.974
CBD	80% (92)	79.8% (95)	
Hilar	20% (23)	20.2% (24)	

IU: Indiana University; MMC: Main Medical Center; RGC: Rockford GE Associates.

CBD: common bile duct.

**Table 2:** Outcomes in patients stented with 10 Fr and 11.5 Fr stents.

	10 Fr (*n* = 115)	11.5 Fr (*n* = 119)	*P* value
Technical success	99.1% (114/115)	97.4% (116/119)	0.37
Baseline bilirubin (mg/dL)	10.7 ± 6.8	12.8 ± 7.6	0.03
Bilirubin decrease (mg/dL)	8.7 ± 6.1*	10.6 ± 6.9*	0.077*
Days to stent failure	149	258	0.16
Stent-related hosp. days (Mean)	1.6 ± 3.5	1.9 ± 3.8	0.50
Stent-related interventions (mean)	0.74 ± 1.5	0.82 ± 1.5	0.66
Median patient survival days	151	206	0.20

*After adjusting for difference in baseline bilirubin.

**Table 3:** Total complications by stent size.

Complication	10 Fr (*n* = 115)		11.5 Fr (*n* = 119)	
	Number (%) of patients	Median time to complication (Days)	Number (%) of patients	Median time to complication (Days)
Death	95 (83%)	120	95 (80%)	170
Death before occlusion	66 (57%)	82.5	66 (55%)	114.5
Stent obstruction (all causes)	39 (34%)	107	38 (32%)	147
Stent occlusion	35 (30%)	110	36 (30%)	147
Stent migration	5 (4%)	61	5 (4%)	56
Tumor overgrowth	1 (1%)	14	3 (3%)	74

**Table 4:** Stent obstruction by stricture location.

Stricture location	10 Fr	11.5 Fr	*P* value
Common duct	28/92 (30%)	26/95 (27%)	0.64
Hilar	11/23 (48%)	12/24 (50%)	0.88

Total	39/115 (34%)	38/119 (32%)	0.75

**Table 5:** Incidence of early (≤30 days) complications.

Complication	10 Fr *n* = 115	11.5 Fr *n* = 119	*P* value
Death	7 (6%)	13 (11%)	0.19
Stent occlusion	6 (5%)	4 (3%)	0.48
Stent migration	2 (2%)	2 (2%)	0.97
Tumor overgrowth	1 (0.9%)	0 (0%)	0.31
Other	1 (0.9%)	4 (3%)	0.19
